# Effects of Resistance Exercise on Neuroprotective Factors in Middle and Late Life: A Systematic Review and Meta-Analysis

**DOI:** 10.14336/AD.2022.1207

**Published:** 2023-08-01

**Authors:** Eva Rodríguez-Gutiérrez, Ana Torres-Costoso, Carlos Pascual-Morena, Diana P Pozuelo-Carrascosa, Miriam Garrido-Miguel, Vicente Martínez-Vizcaíno

**Affiliations:** ^1^Health and Social Research Center, Universidad de Castilla-La Mancha, 16071 Cuenca, Spain.; ^2^Facultad de Fisioterapia y Enfermería, Universidad de Castilla-La Mancha, 45071, Toledo, Spain.; ^3^Grupo de Investigación Multidisciplinar en Cuidados (IMCU), Campus de Fábrica de Armas, Universidad de Castilla-La Mancha, Toledo, 45071, Spain.; ^4^Facultad de Enfermería, Universidad de Castilla-La Mancha, 02006, Albacete, Spain.; ^5^Facultad de Ciencias de la Salud, Universidad Autónoma de Chile, 3460000 Talca, Chile.

**Keywords:** brain-derived neurotrophic factor, insulin-like growth factor type 1, vascular endothelial growth factor, strength exercise, neuroplasticity

## Abstract

Neuroprotective factors are involved in brain functioning. Although physical exercise has been shown to have a positive influence on these factors, the effect of resistance exercise on them is not well known. This systematic review and meta-analysis aimed to 1) estimate the efficacy of resistance exercise on major neuroprotective factors, such as insulin-like growth factor-1 (IGF-1), brain-derived neurotrophic factor (BDNF), and vascular endothelial growth factor (VEGF), in middle and late life and 2) determine whether the effect is dose dependent. A systematic search was conducted in CINAHL, Cochrane CENTRAL, MEDLINE, Scopus, PEDro, SPORTDiscus, and Web of Science up to November 2022. Random effects models were used to estimate standardized mean differences (SMDs) and their respective 95% confidence intervals (CI) for the effect of resistance exercise on peripheral IGF-1, BDNF or VEGF levels in older adults. Thirty randomized clinical trials with 1247 subjects (53.25% women, 45-92 years) were included in the systematic review, and 27 were selected for the meta-analysis. A significant effect of resistance exercise on IGF-1 levels was observed (SMD: 0.48; 95% CI: 0.27, 0.69), being more effective when performing 3 sessions/week (SMD: 0.55; 95% CI: 0.31, 0.79) but not on BDNF (SMD: 0.33; 95% CI: -0.29, 0.94). The effect on VEGF could not be determined due to the scarcity of studies. Our data support the resistance training recommendation in middle and late life, at a frequency of at least 3 sessions/week, to mitigate the neurological and cognitive consequences associated with aging, mainly through IGF-1.

## 1. Introduction

Aging is a natural physiological process associated with cellular and synaptic changes at the brain level related to cognitive processes. Cognitive decline is a slow process that begins in middle life [1] and this is characterized by a loss of volume in the hippocampus, changes in white matter and atrophy and a decrease in gray matter in the prefrontal cortex [2,3], in addition to a reduction in neuroprotective factors [4]. The main substances of a protein nature include insulin-like growth factor type 1 (IGF-1), brain-derived neurotrophic factor (BDNF) and vascular endothelial growth factor (VEGF), which, combined with other hormones and neurotransmitters, have an important role in cell proliferation and growth as well as in neuronal development and function [4-6].

IGF-1 is a peptide that regulates the effects of growth hormones, and BDNF belongs to the neurotrophin family. Both are essential proteins in brain development and tissue remodelling [7,8]. They provide great benefits to cognition due to their effects on neuroplasticity [5,9]. Moreover, these factors are expressed in some regions of the central nervous system specific to cognition, supporting the idea that their decrease causes cognitive impairment [5,8]. Finally, VEGF, like BDNF, is a neurotrophin with some neuroprotective effect [10]. It is an angiogenic factor that has the capacity to preserve brain cells and slow the deterioration of spatial memory and cognitive impairment [10-12]. There is sufficient evidence to support that physical exercise has a positive influence on the release of neuroprotective factors and their cerebral effect by increasing their expression in the central nervous system [13]. Considering that these are peptides that cross the blood–brain barrier, the elevation of their peripheral levels as a consequence of exercise has been reported to favor learning, neurogenesis and angiogenesis [14].

The effect of resistance exercise on these factors has not been sufficiently studied in older adults, and the literature thus far has shown inconclusive results for VEFG [16,17], while they seem to be more consistent for IGF-1[18] and BDNF [19]. On the other hand, the importance of training parameters such as exercise frequency or intensity in enhancing neuroprotective factors has been described [20], although the magnitude of influence of these training characteristics has not been sufficiently quantified. Therefore, the objectives of this systematic review and meta-analysis were 1) to update and synthesize the available evidence regarding the effect of resistance exercise on key neuroprotective factors at the peripheral level in middle and late life and 2) to determine whether the effect depends on exercise dose.

## 2. Methods

The present systematic review and meta-analysis was conducted following the recommendations of the Cochrane Handbook of Systematic Reviews of Interventions [21], and the standards for systematic reviews and meta-analyses of the PRISMA Statement [22] were followed. This review was registered in the PROSPERO database (registration number: CRD420223 02859).

### 2.1 Search strategy

A systematic search was conducted in the following bibliographic databases: CINAHL (via EBSCOhost), Cochrane Central Register of Controlled Trials, MEDLINE (via PubMed), Embase (via Scopus), Physiotherapy Evidence Database (PEDro), SPORTDiscus (via EBSCOhost) and Web of Science from inception up to November 2022. Search terms included "strength exercise", "resistance exercise", "strength training", "resistance training", "weight training", "weightlifting", “IGF-1”, "insulin-like growth factor 1", “BDNF”, "brain-derived neurotrophic factor", “VEGF”, "vascular endothelial growth factor" and “random* control* trials”. The references of the included studies were also reviewed. The search strategy in the different databases can be found in Supplementary Table 1.

In addition, it was necessary to contact four authors [16,23-25], obtaining a response from only one of them because the data required to carry out the meta-analysis could not be obtained from the articles.

The systematic search was performed independently by two reviewers (E.R.G. and A.T.C.). When there were disagreements, a third researcher made the final decision (V.M.V.).

### 2.2 Eligibility criteria

The inclusion criteria for the systematic review and meta-analysis were as follows: 1) participants: adults with a mean age ≥ 45 years; 2) intervention: resistance exercise (minimum 1 session); 3) comparator: control group; and 4) outcome: concentration of BDNF, IGF-1 or VEGF in serum and/or plasma. Furthermore, the exclusion criteria were as follows: 1) studies other than randomized clinical trials (RCTs); 2) subjects with cognitive impairment.

Two independent reviewers (E.R.G. and A.T.C.) conducted the study selection. When there were disagreements, a third researcher made the final decision (V.M.V.).

### 2.3 Data extraction

The full texts of the included studies were reviewed, and the main data were independently extracted from the included studies by 2 reviewers (E.R.G. and A.T.C.) and synthesized in an ad hoc table including 1) author's name, 2) year of publication, 3) country, 4) population characteristics (final number of participants in each group, proportion of women, age of participants, health status), 5) intervention characteristics (type of intervention in each group, frequency, duration, intensity and volume) and 6) outcome (IGF-1, BDNF or VEGF levels in plasma and/or serum). A third reviewer (V.M.V.) was consulted to resolve disagreements between reviewers.

Continuous data were extracted from the studies (including prepost mean IGF-1, BDNF and VEGF values, standard deviation and sample size of the intervention and control groups).

For statistical analysis, all IGF-1, BDNF and VEGF values were transformed to the same unit (ng/mL) (where 1 ng/mL = 1000 pg/mL).

### 2.4 Risk of bias assessment

Two reviewers (E.R.G. and A.T.C.) independently assessed the risk of bias of the included studies using the Cochrane Risk of Bias Tool for Randomized Clinical Trials (RoB 2.0) [21]. Any discrepancies were resolved by a third reviewer (V.M.V.). This tool consists of an assessment based on the following domains: randomization process, deviations from intended interventions, missing outcome data, outcome measurement, and selection of reported outcome. Each of these domains can be assessed as "low risk of bias", "some concerns" and "high risk of bias". Therefore, the overall risk for each of the studies was classified as "low risk of bias" when a low risk of bias was determined for all domains; "unclear risk of bias" when at least one domain had unclear risk but no high risk of bias for any specific domain; and "high risk of bias" when at least one domain was assessed as high risk of bias or as unclear risk of bias in multiple domains [26].

**Figure 1.**
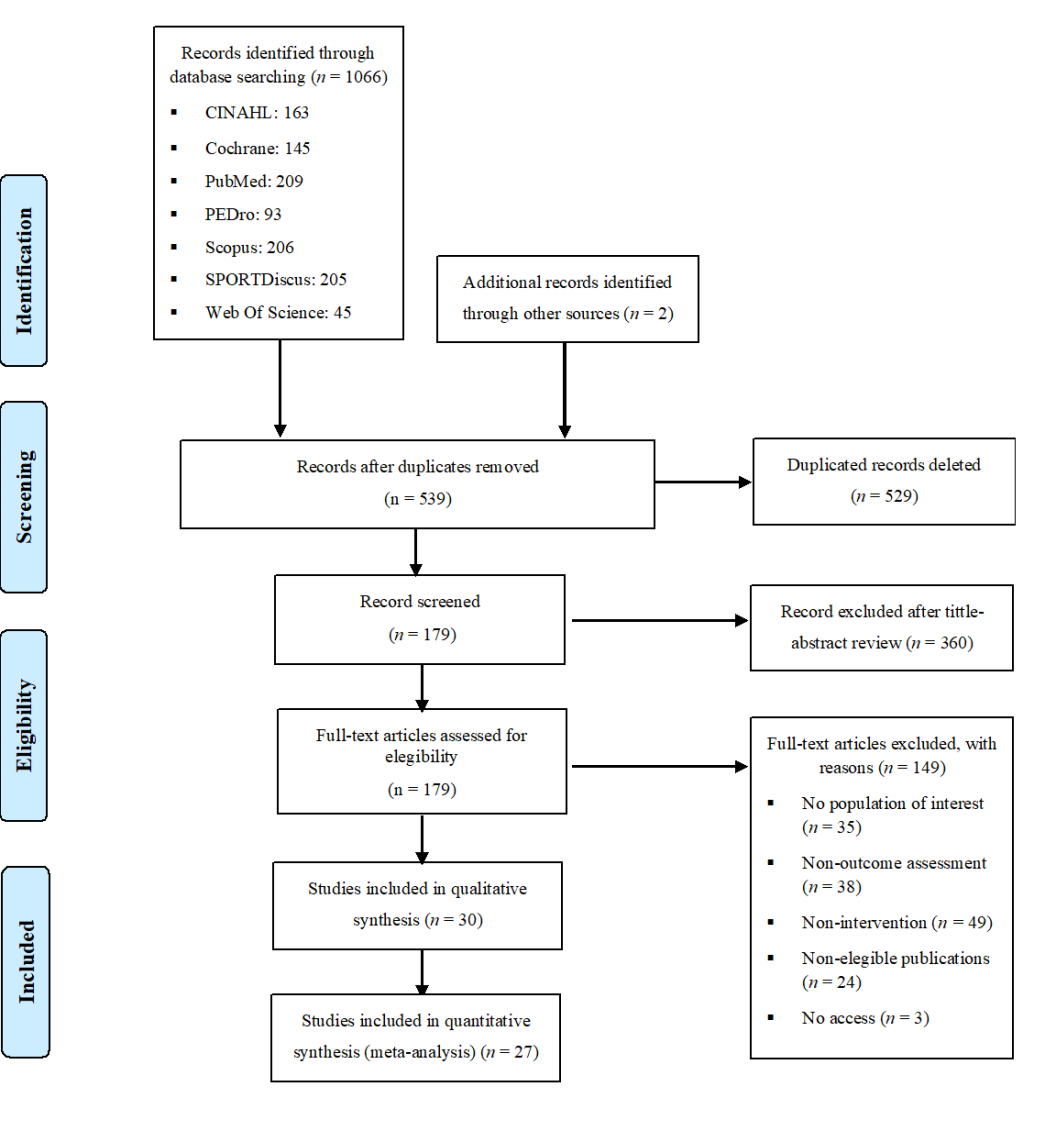
Flow diagram of the studies through the review.

### 2.5 Data analysis

The estimated pooled standardized mean differences (SMDs) of the mean differences for IGF-1, BDNF and VEGF and their 95% confidence intervals (CIs) were calculated using Cohen’s d index [21]. When repeated measurements were reported, we considered only the last measurement as the end point. When a study had two intervention groups that performed resistance exercise, they were taken into account as different studies in the analysis of the results.

A meta-analysis for each factor was performed using a random-effects model with the DerSimonian and Laird method [27] to determine the effect of resistance exercise on neuroprotective factors compared to a control group. Heterogeneity of results between studies was assessed using the I^2^ statistic, which is classified as unimportant (0% to 30%), moderate (30% to 50%), substantial (50% - 75%) and high (75% - 100%). The corresponding p values were also considered [21]. As recommended by the Cochrane Handbook, when data on standard deviation were not reported, they were estimated using the standard error, the CI or a statistical test (t test, F test or a p value) [21].

A sensitivity analysis was performed to determine the robustness of the estimates by eliminating each study included in the meta-analysis one by one, as well as studies in which the population had any specific health disorder or pathology, to determine whether any represented a large proportion of heterogeneity in the pooled ES. For neuroprotective factors where a significant difference was found after resistance exercise, the dose–response relationship was estimated by subgroup analysis according to frequency (days/week), sets, exercise intensity, considering high intensity >10 repetitions maximum (RM)(28) and ≥70% RM [29] and light-moderate intensity ≤10RM and <70% RM and those studies in which indicate that they perform light and/or moderate intensity exercises, the session time (minutes) of the intervention and duration of the exercise program (months). We also conducted a subgroup analysis by sex and a meta-regression model to determine the influence of body mass index and age on this association.

Publication bias was evaluated using Egger's regression asymmetry test, with p values less than 0.10 considered statistically significant. STATA Statistical software, version 16 (StataCorp LLC, College Station, TX, USA) was used to perform the statistical analyses.

## 3. Results

### 3.1 Study selection

The flow chart with the study selection procedure of the systematic review and meta-analysis is shown in detail in Fig. 1. A total of 1066 studies were found (Supplementary Fig. 1), and 179 potentially includable studies were identified through the title and abstract, of which 30 RCTs [17,23-25,30-55] met the eligibility criteria and were included in the systematic review; of these, 27 were included in the meta-analysis [17,23,30-38,40-55] (3 were excluded because no data were available).

### 3.2 Study and intervention characteristics

The most significant characteristics of the studies analysed in this systematic review are shown in Table 1. A total of 30 RCTs [16,23-25,30-48] published between 1994 and 2021 involving 1247 subjects (664 women; 53.25%) aged 45 to 92 years were included in the systematic review. The RCTs were conducted in the following continents: Oceania [39], Asia [17,32,33, 42, 44,48,50], Europe [24,31,41,43,45,46,52] and America [23, 25, 30,34-38,40,47,49,53-55]. In addition, 22 studies were performed in a healthy population, described as individuals without specific health disorders or pathologies [23,24,30-32,34-38,40,42-47,49-51,53,55] and 8 in populations suffering from specific diseases or health disorders such as sarcopenic obesity [33], type 2 diabetes [39], limited mobility [41], low bone mineral density [25], hypertension [48], coronary heart disease [17], chronic kidney disease [54] and fibromyalgia [52].

Resistance training ranged from 1 to 6 sets and 5 to 25 repetitions at 30-85% one repetition maximum (1RM). The length of the exercise programs was up to 12 months and the frequency ranged from 2 to 3 days/week, prevailing 3 days/week. In most studies, the control group did not exercise. In three studies, they performed warm-up and/or stretching [34,35,39], and in one, they performed cognitive training [45] (Table 1). IGF-1 was analysed in 23 articles [23-25,30,32-36,38-40,42,44-46,48-53,55], in 2 measured through its concentration in plasma [46,48], in 16 through serum [23, 30, 32-35, 38, 40,42,44,45,50-53,55] and the remaining 5 did not reported these data [24,25,36,39,49]; BDNF was analysed in 7 articles [23,24,32,33,37,45,54], in all of them, except one, through serum [31]; finally, VEGF was only measured in one article through its concentration in serum with significant results [17].

### 2.3 Risk of bias assessment

According to the RoB 2.0. tool [26], 11 out of 30 were classified as "high risk of bias", and 19 out of 30 were classified as "unclear risk of bias". The most affected domains were randomization process, deviations from intended interventions and selection of the reported result. The assessment of risk is shown in Supplementary Fig. 2.

### 2.4 Meta-analysis

**Figure 2.**
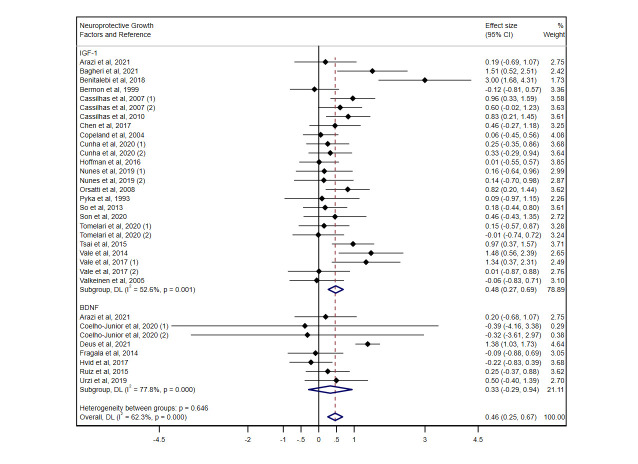
**Standardized mean difference (95% CI) of the effect of resistance exercise vs**. the control group on neuroprotective factors after intervention, with subgroup analysis by factors.

The pooled SMD of resistance exercise on IGF-1 levels was 0.48 (95% CI: 0.27, 0.69; I^2^ = 52.6%, *P*=0.001) (Fig. 2). In relation to training characteristics, exercise frequency showed significant results with at least 3 days per week (SMD: 0.55; 95% CI: 0.31, 0.79). In relation to the number of sets of the program, both performing 2 or fewer and more than 2 sets showed significant benefits, as well as light-moderate and high-intensity exercises, sessions of more or less than 60 minutes and exercise programs of more or less than 3 months (Table 2). In both men and women, IGF-1 levels increased significantly after resistance exercise (Supplementary Table 2). Meta-regression analysis showed that neither body mass index nor age influenced peripheral IGF-1 levels (*P*>0.05) in older adults (Supplementary Table 3).

BDNF levels were not significantly different when performing resistance exercise versus the control group (SMD: 0.33; 95% CI: -0.29, 0.94; I^2^ = 77.8%, *P*=0.000) (Fig. 2). Subgroup analyses on sex could not be performed due to the limited number of studies.

VEGF was only analysed by one study [17], so it was not included in the meta-analysis. Significant differences were observed between the resistance exercise group and the control group.

**Table 1:** General characteristics of the studies

Study Characteristics		Population characteristics			Intervention characteristics				
**Author, year**	Country	n (female)	Age, years	Health status	Intervention	Frequency	Duration	Intensity and volume	Outcome
**Arazi et al, (2021) (29)**	Iran	IG-1: 10 (0) IG-2: 10 (0) CG: 10 (0)	IG-1: 60.8 ± 1.8 IG-2: 60.7 ± 1.7 GC: 60.9 ± 0.9	Healthy	IG-1: resistance exercise IG-2: aerobic exercise CG: no exercise	1 day	1 day	45 min, 2 sets x 10 rep, 65-70% 1RM	BDNF IGF-1 (serum)
**Bagheri et al (2021) (51)**	NR	IG: 10 (10) CG: 10 (10)	56 ± 3.7	Healthy, Sedentary	IG: resistance exercise CG: no exercise	3 days/ week	8 weeks	3-4 sets x 10-12 rep, 60-75% 1RM	IGF-1 (serum)
**Benitalebi, et al (2020) (50)**	Iran	IG: 10 (10) CG: 9 (9)	67.35 ± 1.4	Healthy	IG: resistance exercise CG: no exercise	3 days/ week	12 weeks	50 min, 2-3 sets x 8-16 rep, 40-75% 1RM	IGF-1 (serum)
**Bermon et al, (1999) (43)**	France	IG: 16 (NR) CG: 16 (NR)	IG: 70.1 ± 1 CG: 70.5 ± 0.9	Healthy, Sedentary	IG: resistance exercise CG: no exercise	3 days/ week	8 weeks	3 sets x 8 rep, 80% 1RM	IGF-1 (plasma)
**Cassilhas et al, (2010) (32)**	Brazil	IG: 20 (0) CG: 23 (0)	IG: 68.4 ± 0.67 CG: 67.04 ± 0.54	Healthy, Sedentary	IG: resistance exercise GC: warm-up and stretching	3 days/ week	24 weeks	1 hour, 2 sets x 8 rep, 80% 1RM	IGF-1 (serum)
**Cassilhas et al, (2007) (31)**	Brazil	IG-1: 20 (0) IG-2: 19 (0) CG: 23 (0)	IG-1: 68.4 ± 0.67 IG-2: 69.01 ± 1.1 CG: 67.04 ± 0.54	Healthy, Sedentary	IG-1: high intensity resistance exercise IG-2: low intensity resistance exercise CG: warm-up and stretching	3 days/ week	24 weeks	IG-1: 1 hour, 2 sets x 8 rep, 80% 1RM. IG-2: 1 hour, 2 sets x 8 rep, 50% 1RM.	IGF-1 (serum)
**Chen et al, (2017) (30)**	China	IG-1: 15 (12) IG-2: 15 (14) IG-3: 15 (11) CG: 15 (13)	IG-1: 68.9 ± 4.4 IG-2: 69.3 ± 3.0 GI-3: 68.5 ± 2.7 GC: 68.6 ± 3.1	Sarcopenic obesity	IG-1: resistance exercise IG-2: aerobic exercise IG-3: combined exercise (resistance + aerobic) CG: no exercise	2 days/ week	12 weeks	IG-1: 1 hour, 3 sets x 8-12 rep, 60-70% 1RM. IG-2: 1-hour, different exercises of moderate intensity. IG-3: Each day they perform a different exercise program.	IGF-1 (serum)
**Chen et al, (2019) (17)**	China	IG-1: 19 (8) CG: 18 (7)	IG-1: 62.84 ± 5.54 CG: 65.89 ± 5.51	Coronary heart disease	IG: isometric resistance exercise CG: no exercise	2 sessions per day, 5 days/ week	3 months	1 set x 10 rep, 40-50% 1RM	VEGF (serum)
**Coelho-Junior et al, (2020) (44)**	Brazil	IG-1: 10 (10) IG-2: 12 (12) CG: 14 (14)	IG-1: 67 ± 6.2 GI-2: 66.7 ± 5.1 GC: 66.7 ± 4.6	Healthy	IG-1: traditional resistance exercise IG-2: resistance-power exercise with an elastic band CG: no exercise	2 days/ week	22 weeks	IG-1: 1 hour, 3 sets x 8-10 rep, intensity 5-6 (Borg scale). IG-2: 1 hour, 3 sets x 8-10 rep at intensity 3 (Borg scale) performing concentric phase as fast as possible.	BDNF (serum)
**Copeland et al, (2014) (55)**	Canada	IG-1: 16 (16) CG: 16 (16)	IG-1: 53.8 ± 5.85 CG: 56.6 ± 5.6	Healthy, Sedentary	IG: resistance exercise CG: no exercise	3 days/ week	12 weeks	2-3 sets x 10 rep, 10RM	IGF-1 (serum)
**Cunha et al, (2020) (33)**	Brazil	IG-1: 21 (21) IG-2: 20 (20) CG: 21 (21)	IG-1: 70.09 ± 5.95 GI-2: 68.6 ± 4.44 GC: 68.04 ± 4.38	Healthy	IG-1: resistance exercise IG-2: resistance exercise CG: no exercise	3 days/ week	12 weeks	IG-1: 15 min, 1 set x 10-15RM IG-2: 45 min, 3 sets x 10-15RM	IGF-1 (NR)
**Daly et al, (2005) (36)**	Australia	IG: 14 (NR) CG: 12 (NR)	IG: 67.6 ± 5.2 GC: 66.9 ± 5.3	Diabetes type 2, Sedentary, Overweight	IG: resistance exercise CG: stretching	3 days/week	12 months	55 min, 3 sets x 8-10 rep, 75-85% 1RM (1-6 months) and 60-80% 1RM (6-12 months at home).	IGF-1 (NR)
**Deus et al, (2021) (54)**	Brazil	IG: 81 (NR) CG: 76 (NR)	IG: 67.27 ± 3.24 GC: 66.33 ± 3.88	Chronic kidney disease	IG: resistance exercise CG: no exercise	3 days/ week	6 months	1 hour, 3 sets, 8-12 rep	BDNF (serum)
**Fragala et al, (2014) (34)**	United States	IG: 13 (NR) CG: 12 (NR)	IG: 70.64 ± 6.11 GC: 70.64 ± 6.11	Healthy	IG: resistance exercise CG: no exercise	2 days/ week	6 weeks	3 sets x 8-15 rep, moderate intensity (5-6 OMNI scale)	BDNF (serum)
**Hofmann et al, (2016) (42)**	Austria	IG-1: 26 (26) IG-2: 21 (21) CG: 23 (23)	GI-1: 82.9 (71.7 - 92.2) GI-2: 83.9 (65 - 92.2) GC: 84.5 (69.4 - 91.8)	Healthy	IG-1: resistance exercise IG-2: resistance exercise + nutrition CG: cognitive training	2 days/week	6 months	IG-1 y IG-2: 60 min, 1-2 sets x 15rep, adapting the resistance of the elastic band. CG: memory and coordination activities	IGF-1 (serum)
**Hvid et al, (2017) (38)**	Denmark	IG: 20 (NR) CG: 22 (NR)	IG: 82.7 ± 5.4 CG: 82.2 ± 4.5	Limited mobility	IG: resistance-power exercise CG: no exercise	2 days /week	12 weeks	3 sets x 8-10 rep, 70-80% 1RM performing concentric phase as fast as possible.	BDNF (serum)
**Nunes et al, (2019) (49)**	Brazil	IG-1: 12 (12) IG-2: 10 (10) CG: 12 (12)	IG-1: 59.7 (55.9 - 63.5) IG-2: 64.2 (58.4 - 69.9) CG: 59.0 (55.4 - 62.5)	Healthy	IG-1: resistance exercise IG-2: resistance exercise CG: no exercise	3 days/ week	16 weeks	IG-1: 90 min, 3-6 sets x 8.12 rep, 70% 1RM IG-2: 45 min, 3 sets x 8.12 rep, 70% 1RM	IGF-1 (NR)
**Orsatti et al, (2008) (53)**	Brazil	IG: 21 (21) CG: 22 (22)	IG: 57.8 ± 8.0 CG: 59.3 ± 6.2	Healthy, Sedentary	GI: resistance exercise CG: no exercise	3 days/ week	16 weeks	50-60 min, 1-3 sets, 8-15 rep, 60-80% 1RM	IGF-1 (serum)
**Parkhouse et al, (2000) (24)**	Canada	IG: 13 (13) CG: 9 (9)	IG: 67 ± 1 GC: 70 ± 2	Low bone mineral density, Sedentary	GI: resistance exercise CG: no exercise	3 days/ week	8 months	3 sets x 8-10 rep, 75-80% 1RM	IGF-1 (serum)
**Pyka et al, (1993) (22)**	United States	IG: 8 (NR) CG: 6 (NR)	GI: 69.6 ± 1.1 GC: 69.6 ± 1.1	Healthy	IG: resistance exercise CG: no exercise	3 days/ week	52 weeks	3 sets x 8 rep, 65-75% 1RM	IGF-1 (serum)
**Ruiz et al, (2015) (40)**	Spain	IG: 20 (16) CG: 20 (16)	IG: 92.3 ± 2.3 CG: 92.1 ± 2.3	Healthy	IG: resistance exercise CG: no exercise	3 days/ week	8 weeks	40-45 min, 1-3 sets x 8-10 rep, 30-70% 1RM	BDNF (serum)
**Sartorio et al, (2015) (23)**	Italy	IG: 16 (0) CG:14 (0)	IG: 72.9 ± 0.95 CG: 73.3 ± 1.04	Healthy	IG: resistance exercise CG: no exercise	3 days/ week	16 weeks	6 sets x 10 rep, 50-80% 1RM (MMII) and 40-65% (MMSS).	IGF-1 (NR)
**So et al, (2013) (39)**	South Korea	IG: 18 (12) CG: 22 (15)	IG: 71.6 ± 5.5 CG: 68.4 ± 5.8	Healthy	IG: resistance exercise with elastic bands CG: no exercise	3 days/ week	12 weeks	60 min, 2-3 sets x 15-25 rep with red elastic band (low intensity).	IGF-1 (serum)
**Son et al, (2020) (45)**	South Korea	IG: 10 (10) CG: 10 (10)	IG: 67.8 ± 1.1 CG: 67.6 ± 1.3	Stage 1 hypertension, Sedentary	IG: resistance exercise CG: no exercise	3 days/week	12 weeks	60 min, 2-4 sets x 10-20 rep, 40-70% 1RM.	IGF-1 (plasma)
**Tomelari et al, (2020) (37)**	Brazil	IG-1: 15 (15) IG-2: 14 (14) CG: 15 (15)	GI-1: 71.4 ± 6 GI-2: 69.7 ± 5.7 CG: 68.6 ± 5.1	Healthy	IG-1: mono-joint resistance exercise IG-2: multijoint resistance exercise CG: no exercise	3 days/ week	12 weeks	3 sets x 10-15RM.	IGF-1 (serum)
**Tsai et al, (2015) (41)**	China	IG: 21 (0) CG: 20 (0)	IG: 66.05 ± 6.64 CG: 64.5 ± 6.95	Healthy	IG: resistance exercise CG: no exercise	3 days/ week	12 months	60 min, 3 sets x 10 rep, 75-80% 1RM.	IGF-1 (serum)
**Urzi et al, (2019) (28)**	Eslovenia	IG: 11 (11) CG: 9 (9)	IG: 84.4 ± 7.7 CG: 88.9 ± 5.3	Healthy	IG: resistance exercise CG: no exercise	3 days/ week	12 weeks	45-50 min, 1-3 sets x 5-12 rep, mild-moderate intensity.	BDNF (plasma)
**Vale et al, (2017) (27)**	Brazil	IG-1: 10 (10) IG-2: 10 (10) CG: 10 (10)	GI-1: 66.1 ± 2.77 IG-2: 67.1 ± 3.54 GC: 68.8 ± 5.41	Healthy	IG-1: resistance exercise on land IG-2: resistance exercise in water CG: no exercise	3 days/ week	12 weeks	IG-1: 50 min, 3 sets x 15 rep, 50% 1RM (1-4 week) y 3 sets x 8-10 rep, 75-85% 1RM (5-12 week). IG-2: 50 min, 3 sets x 15-20 rep without aquatic accessories (1-4 week) y 3 sets x 8-10 rep aquatic accessories (5-12 week).	IGF-1 (serum)
**Vale et al, (2014) (35)**	Brazil	IG: 14 (14) CG: 10 (10)	IG: 69 ± 5.1 CG: 69 ± 5.9	Healthy	IG: resistance exercise CG: no exercise	3 days/ week	20 weeks	50 min, 2-3 sets x 15-20 rep, mild-moderate intensity (3-5 on OMNI-RES scale)	IGF-1 (serum)
**Valkeinen et al, (2005) (52)**	Finland	IG: 13 (13) CG: 13 (13)	IG: 60 ± 2 CG: 59 ± 4	Fibromyalgia	IG: resistance exercise CG: no exercise	2 days/ week	21 weeks	10-20 rep, 40-80% 1RM	IGF-1 (serum)

Abbreviations: BDNF = brain-derived neurotrophic factor, CG = control group, IG = intervention group, IGF-1 = insulin-like growth factor type 1, min = minutes, MMII = lower limbs, MMSS = upper limbs, NR = No reported, OMNI-RES = OMNI-Resistance Exercise Scale, rep = repetition, RM = maximum repetition, VEGF = vascular endothelial growth factor

### 3.5 Sensitivity analysis

The pooled ES estimates for the effect of resistance exercise on IGF-1 and BDNF were not significantly changed in magnitude or direction when removing each study included in the meta-analysis one by one, as well as when eliminating studies in which the population had a specific health disorder or pathology.

### 3.6 Publication bias

There was evidence of publication bias, as seen in the funnel plots and Egger's tests for IGF-1 (*P*=0.052) and BDNF (*P*=0.100) (Supplementary Fig. 3).

## 4. Discussion

Although several studies have reported the effectiveness of physical exercise on the main neuroprotective factors, there was no updated review comparing the effect of resistance exercise on these factors and determining the dose necessary to achieve this effect in middle and late life. Our data support that resistance exercise increases IGF-1 levels, being more effective when performed at least three sessions per week, independent of the number of sets, the intensity of the exercise, the time of the session and the duration of the exercise program. However, no significant effects were estimated for peripheral BDNF levels, and in the case of VEGF, the scarce number of articles made it impossible to analyse the effect on this factor.

The progressive decline in IGF-1 levels experienced by the population [56,57] is associated with impaired brain function and risk of vascular dementia [56,58]. The available evidence seems to indicate that resistance exercise could mitigate these adverse effects [18,59]. Several factors could justify the effect of resistance exercise on IGF-1 levels, such as the fact that this type of exercise induces anabolic hormonal responses, allowing the direct release of IGF-1 by the liver or indirectly when induced by growth hormone [57]. Furthermore, a positive relationship has been established between IGF-1 levels, strength, and muscle mass because this factor is capable of increasing the proliferation capacity of muscle satellite cells, thereby preventing the loss of muscle mass related to aging [60].

Regarding the dose necessary to achieve this beneficial effect, our data corroborate that three or more sessions per week would be necessary to increase IGF-1 levels, independent of the number of series, the intensity of the exercise, the duration of the sessions or the duration of the exercise program. However, contrary to what has been reported in previous reviews [18,59], which indicated that the increase in IGF-1 levels would only occur in women, our data show that this association is also positive in men, which seems logical considering that as a consequence of exercise, there are increases in growth hormone levels in both sexes, and this is closely related to IGF-1 synthesis [61].

**Table 2:** Subgroup analysis of the effect of resistance exercise dose (frequency, sets, intensity, session time and duration of exercise program) on IGF-1

Subgroup	n	SMD (95% CI)	I^2^	p
*Frequency of exercise*				
< 3 days	4	0.13 (-0.22, 0.48)	0.0%	0.750
≥ 3 days	21	0.55 (0.31, 0.79)	56.4%	0.001
*Sets of exercises*				
≤ 2 sets	6	0.48 (0.16, 0.79)	30.9%	0.204
> 2 sets	18	0.52 (0.24, 0.80)	59.2%	0.001
*Intensity*				
Light-moderate	11	0.31 (0.11, 0.52)	0.0%	0.475
High	14	0.62 (0.26, 0.97)	65.8%	0.000
*Session time*				
< 60 min	8	0.73 (0.18, 1.28)	71.2%	0.001
≥ 60 min	10	0.56 (0.33, 0.79)	17.7%	0.280
*Duration of exercise program*				
≤ 3 months	15	0.38 (0.10, 0.67)	55.7%	0.005
> 3 months	12	0.53 (0.25, 0.88)	42.1%	0.061

Abbreviations: SMD = standardized mean differences

Different meta-analyses have evaluated the effects of exercise interventions on BDNF, reporting significant effects in adolescents and children and in neurodegenerative disorders [62-64]. In older adults, Marinus et al. [19] evaluated the impact of resistance exercise on BDNF before and after exercise, observing a significant increase in peripheral levels of this neuroprotective factor. However, our results do not show this effect when compared against a control group, which could be because the BDNF generated in skeletal muscle with contraction is not released into the circulation but would be used to enhance muscle oxidation [65]. This could also be because the release and synthesis of this factor occurs immediately after exercise [66]. However, when exercise ceases, this effect disappears, and the peripheral concentration of BDNF normalizes [65,67], which could indicate that circulating BDNF would be transported to the brain through the blood circulation, where it would cross the blood–brain barrier, achieving greater neuronal survival and synaptogenesis and, therefore, greater brain function and structural changes [66].

Moreover, several studies have shown that aerobic exercise has contradictory effects on VEGF [68,69]. Resistance exercise seems to be effective in the adult population when it is performed with blood flow restriction [70]. However, the lack of studies has not allowed a synthesis of the effect of resistance exercise on this factor.

Some limitations of our systematic review and meta-analysis should be acknowledged. First, the lack of studies evaluating the different neuroprotective factors, particularly VEGF, limited the possibility of determining the effect of resistance exercise on this factor. Second, and in relation to the methodological quality of the studies, although in general the quality of the studies was acceptable, a large proportion of them did not provide information about some domains of the RoB 2.0., and the risk of bias was rated as high risk or some concerns. However, to overcome these limitations, sensitivity analyses were performed by eliminating each study included in the meta-analysis one by one. This same process was carried out with those studies in which the population had some pathology or health disorder to provide evidence of the robustness of the results. Third, there was a great heterogeneity of the interventions in terms of type of exercises, volume, frequency, and intensity; however, they have been pooled in a way that can provide conclusive results in relation to the dose–response.

## 5. Conclusion

Our data support a neuroprotective effect of resistance exercise in middle and late life, mainly through its influence on IGF-1. Therefore, physical activity programs targeted to this population should emphasize the promotion of this type of training, with a frequency of at least 3 days/week, to mitigate the neurological and cognitive consequences associated with aging. Because of the scarcity of studies, more clinical trials are needed to consistently establish the neuroprotective effect of resistance exercise, particularly in neuroprotective factors that have not yet been sufficiently studied.

## Supplementary Materials

The Supplementary data can be found online at: www.aginganddisease.org/EN/10.14336/AD.2022.1207.
