# Supplementary material for: Effects of Resistance Exercise on Neuroprotective Factors in Middle and Late Life: A Systematic Review and Meta-Analysis
Source: Aging Dis. 2023 Aug 1;14(4):1264–75. doi: 10.14336/AD.2022.1207 (PMC10389831; doi:10.14336/AD.2022.1207)
Supplement: Supplementary file 1 — The Supplementary data can be found online at: www.aginganddisease.org/EN/10.14336/AD.2022.1207. [file AD-14-4-1264-s.pdf]

SUPPLEMENTARY DATA

Effects of Resistance Exercise on Neuroprotective Factors in Middle and Late Life: A Systematic Review and Meta-Analysis

Eva Rodríguez-Gutiérrez, Ana Torres-Costoso, Carlos Pascual-Morena, Diana P Pozuelo-Carrascosa, Miriam Garrido-Miguel, Vicente Martínez-Vizcaíno

© 2022. Rodríguez-Gutiérrez E. et al. Published online at https://www.aginganddisease.org/EN/10.14336/AD.2022.1707

SUPPLEMENTARY DATA

Supplementary Table 1. Database search strategy.

Database	Search strategy
CINAHL	("strength exercise" OR "resistance exercise" OR "strength training" OR "resistance training" OR "weight training" OR "weight lifting") AND (IGF-1 OR "insulin-like growth factor 1" OR BDNF OR "brain-derived neurotrophic factor" OR VEGF OR "vascular endothelial growth factor") AND (random* control* trials) NOT (animal OR rat OR mouse)
Cochrane	
MEDLINE	
SPORTDiscus	
Scopus	
Web of Science	
PEDro	strength training IGF-1 randomized clinical trial<br>resistance training IGF-1 randomized clinical trial<br>strength training BDNF randomized clinical trial<br>resistance training BDNF randomized clinical trial<br>strength training VEGF randomized clinical trial<br>resistance training VEGF randomized clinical trial

Abbreviations: BDNF = brain-derived neurotrophic factor, IGF-1 = insulin-like growth factor type 1, VEGF = vascular endothelial growth factor

Supplementary Table 2. Subgroup analysis of the effect of resistance exercise on IGF-1 by sex.

Subgroup	n	SMD (95% CI)	I^2	p
Man	5	0.77 (0.48, 1.06)	0.0%	0.599
Woman	16	0.49 (0.18, 0.79)	61.6%	0.001

Abbreviations: CI = confidence interval, SMD = standardized mean differences, IGF-1 = insulin-like growth factor type 1

Supplementary Table 3. Meta-regression analysis of the effect of resistance exercise versus control group on IGF-1 levels.

	n	*Age ß (95% CI)*	p	n	*BMI ß (95% CI)*	p
**IGF-1**	24	-0.288<br>(-0.866, 0.291)	0.313	24	-0.011<br>(-0.054, 0.033)	0.619

BMI Body mass index, IGF-1 Insulin-like growth factor type 1

© 2022. Rodríguez-Gutiérrez E. et al. Published online at https://www.aginganddisease.org/EN/10.14336/AD.2022.1707

SUPPLEMENTARY DATA

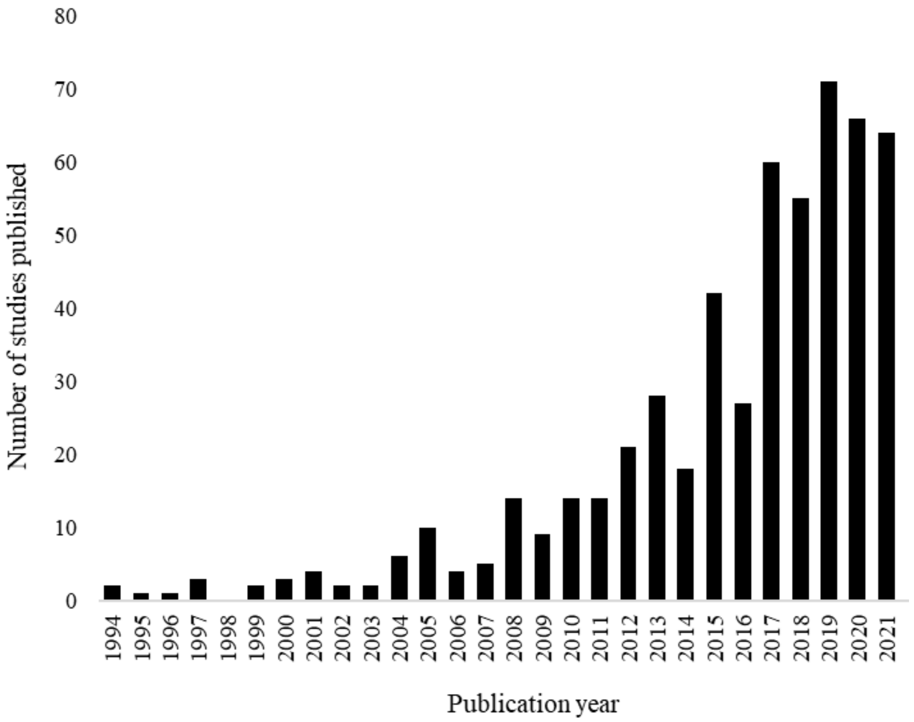

Supplementary Figure 1. Number of studies published per year.

© 2022. Rodríguez-Gutiérrez E. et al. Published online at https://www.aginganddisease.org/EN/10.14336/AD.2022.1707

SUPPLEMENTARY DATA

	Randomization process	Deviations from intended interventions	Missing outcome data	Measurement of the outcome	Selection of the reported result	Overall Bias
Arazi et al, 2020	+	?	+	+	?	!
Bermon et al, 1999	?	?	+	+	?	!
Bagheri et al, 2021	?	+	+	+	?	!
Benitalebi et al, 2020	+	+	+	+	?	!
Casshilas et al, 2010	?	?	+	+	?	!
Casshilas et al, 2007	?	?	+	?	?	!
Chen et al, 2017	?	„	?	+	?	„
Chen et al, 2019	+	?	+	?	?	!
Coelho-Junior et al, 2020	+	„	+	+	+	„
Copeland et al, 2014	?	+	+	+	?	!
Cunha et al, 2020	+	?	+	+	?	!
Daly et al, 2005	?	„	?	+	?	„
Deus et al, 2021	+	+	+	+	?	!
Fragala et al, 2014	?	?	+	+	?	!
Hofmann et al, 2016	?	„	?	+	?	„
Hvid et al, 2017	?	„	+	+	+	„
Nunes et al, 2019	?	+	+	+	?	!
Orsatti et al, 2008	?	+	+	+	?	!
Parkhouse et al, 2010	„	?	+	+	?	„
Pyka et al, 1994	?	?	+	+	?	!
Ruiz et al, 2015	?	+	+	+	?	!
Sartorio et al, 2001	?	?	+	+	?	!
So et al, 2013	?	„	+	+	?	„
Son et al, 2020	?	+	+	+	?	!
Tomelari et al, 2020	?	„	+	+	?	„
Tsai et al, 2015	?	?	„	+	?	!
Urzi et al, 2019	?	„	?	+	?	„
Vale et al, 2017	?	„	+	+	?	„
Vale et al, 2014	?	„	+	?	?	„
Valkeinen et al, 2005	?	+	+	+	?	!

+ Low risk
? Some concerns
„ High risk

Supplementary Figure 2. Risk of Bias (RoB 2.0).

© 2022. Rodríguez-Gutiérrez E. et al. Published online at https://www.aginganddisease.org/EN/10.14336/AD.2022.1707

SUPPLEMENTARY DATA

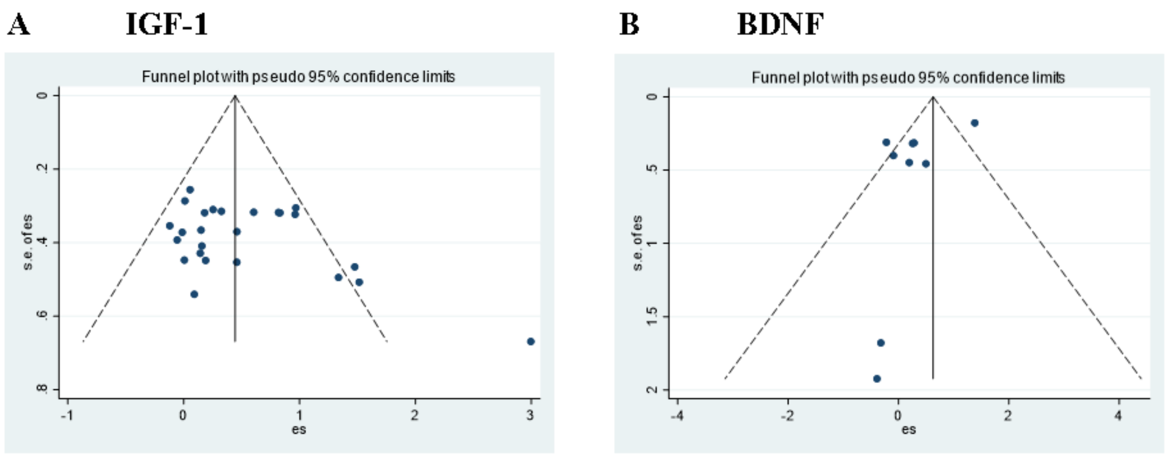

Supplementary Figure 3. Funnel plot with 95% pseudo confidence limits for: A. IGF-1; B. BDNF.

© 2022. Rodríguez-Gutiérrez E. et al. Published online at https://www.aginganddisease.org/EN/10.14336/AD.2022.1707
